# The KOALA-study: study protocol for a comprehensive study of cognitive biases in adolescent anorexia nervosa patients compared to healthy and clinical controls

**DOI:** 10.1186/s40337-021-00494-6

**Published:** 2021-10-29

**Authors:** Anca Sfärlea, Linda Lukas, Gerd Schulte-Körne, Belinda Platt

**Affiliations:** Department of Child and Adolescent Psychiatry, Psychosomatics and Psychotherapy, University Hospital, Ludwig-Maximilians-University Munich, Pettenkoferstr. 8a, 80336 Munich, Germany

**Keywords:** Anorexia nervosa, Adolescence, Attention bias, Interpretation bias, Memory bias, Scrambled sentences task (SST)

## Abstract

**Background:**

Anorexia nervosa (AN) is characterized by dysfunctional cognitions including cognitive biases at various levels of information processing. However, less is known about the specificity of these biases, i.e., if they occur for eating-disorder-related information alone or also for non-eating-disorder-related emotional information in AN patients (content-specificity) and if they are unique to individuals with AN or are also shown by individuals with other mental disorders (disorder-specificity).

**Methods:**

The present study systematically assesses cognitive biases in 12–18-year-old female adolescents with AN on three levels of information processing (attention, interpretation, and memory) and with regard to two types of information content (eating-disorder-related, i.e., stimuli related to body weight and shape, and non-eating-disorder-related). To address not only content- but also disorder-specificity, adolescents with AN will be compared not only to a healthy control group but also to a clinical control group (adolescents with major depression or particular anxiety disorders). Cognitive biases are assessed within a single experimental paradigm based on the Scrambled Sentences Task. During the task eye movements are recorded in order to assess attention biases while interpretation biases are derived from the behavioural outcome. An incidental free recall test afterwards assesses memory biases. We expect adolescents with AN to show more pronounced negative cognitive biases on all three levels of information processing and for both types of content compared to healthy adolescents. In addition, we expect the specificity of biases to translate into differential results for the two types of content: AN patients are expected to show stronger biases for disorder-related stimuli but similar or less pronounced biases for non-disorder-related stimuli compared to the clinical control group.

**Discussion:**

This is the first study to comprehensively assess cognitive biases in adolescents with AN. It will have essential implications not only for cognitive-behavioural models of AN but also for subsequent studies aiming to modify cognitive biases in this population, thereby addressing important maintaining factors already at an early stage of the disorder.

## Background

Anorexia nervosa (AN) is a mental disorder characterized by significantly low body weight, intense fear of gaining weight and body image disturbance [1]. The disorder primarily affects adolescent girls and women with a lifetime prevalence of between 0.9% and 2.2% [2, 3]. Onset of the disorder is often in adolescence and young adulthood: roughly 40% of patients are 15–19 years old [3]. AN is a severe and highly debilitating disorder, with the highest mortality rate of all mental disorders [4, 5] and a relatively poor long-term prognosis: less than half of patients recover completely and about 20% of cases take a chronic course [6, 7].

In addition to the core symptoms, patients with AN often show dysfunctional cognitions regarding disorder-related information, i.e., information related to food, body, and weight [e.g., 8–10]. These dysfunctional cognitions manifest themselves, for example, as cognitive biases at different levels of information processing: attention to, interpretation of, and memory for eating disorder (ED)-related information. Attention biases are defined as tendencies to focus attention on information that is consistent with one’s dysfunctional cognitions [e.g., 11]. For example, to attend more to one’s unattractive body parts rather than the garment itself when trying on a new dress. Negative interpretation biases are tendencies to attribute negative rather than neutral or positive meanings to ambiguous information [e.g., 12]. For example, interpreting the shop assistant’s suggestion to try on another dress as “you’re too fat for that dress” instead of “another colour might suit you better”. Memory biases, in turn, refer to tendencies to remember information consistent with one’s dysfunctional cognitions better than other information [e.g., 13], e.g., remembering situations in which someone said something negative about one’s weight or shape better than situations in which one received a compliment. These cognitive biases are not only correlates of the disorder but are considered to play an important role in both the development and maintenance of AN [e.g., 8–11].

Empirical studies have been conducted to examine disorder-specific cognitive biases in AN, mostly focusing on attention biases for different kinds of ED-related information. A well-established finding in patients with AN is that they show attention biases for images or words related to food and eating [see e.g., 11, 14–17, for reviews and meta-analyses] as well as for stimuli related to body weight and shape [see e.g., 11, 15–17]. Interpretation biases have been less frequently examined in AN. Using an Ambiguous Scenarios Task, a negative body-related interpretation bias has been found in patients with AN [18] as well as in a broader ED sample [19]. Similarly, Brockmeyer et al. [12] found a more pronounced negative body-related interpretation bias in patients with AN compared to healthy controls using a Scrambled Sentences Task (SST). There are also only few studies on memory biases in AN. One study found patients with AN to show an explicit but no implicit memory bias for ED-related words [13] whereas another reported evidence of an implicit but no explicit memory bias [20]. Tekcan et al. [21], on the other hand, found a memory bias in AN patients in the form of hampered forgetting of ED-related words.

In addition to these cognitive biases for ED-related information, it has more recently been suggested that individuals with AN also show cognitive biases for non-ED-related emotional information, as for example emotional faces, words, or situations. Studies investigating attention biases for non-ED-related emotional information in AN have produced mixed findings: While some found stronger attention biases *towards* faces showing negative emotions in AN patients compared to healthy controls [22–24], others found AN patients to turn their attention *away* from negative faces [25] or did not find differences in attention biases for emotional faces [26, 27] or words [28] between AN patients and healthy participants. A study that investigated non-ED-related interpretation biases in AN [29] found AN patients to interpret ambiguous social situations more negatively than the control group, i.e., to show a non-ED-specific negative interpretation bias. These more general, non-ED-specific negative biases are similar to those found in individuals suffering from other mental disorders as depression and anxiety [e.g., 30]. Their presence in individuals with AN conflicts with the hypothesis that ED patients are characterized particularly by content-specific, i.e., ED-related, cognitive biases [10]. However, to properly address the content-specificity hypothesis it is necessary to assess biases for both ED-related and non-ED-related information within one study instead of examining biases for the different types of content independently. Only few studies have done so: two studies of attention biases in patients with AN and other EDs that used both ED-related and non-ED-related words found biases only for the ED-related words [31, 32]. Similarly, Williamson et al. found interpretation biases only for ED-related but not for non-ED-related ambiguous situations [19] and Hermans et al. found memory biases only for ED-related but not for non-ED-related negative words in AN patients [13].

In summary, there remains a lot to be known about cognitive biases in AN. There is evidence of biases for ED-related information on all three levels of information processing and, with regard to attention and interpretation biases, also for non-ED related information. However, particularly regarding interpretation and memory biases, evidence is rather scarce and the comparability of the studies and aggregation of their results is limited due to the use of different methods and different types of stimuli for measuring biases. The content-specificity hypothesis [10] has rarely been addressed [13, 19, 31–33]. Furthermore, no study has examined biases on all three levels of information processing within the same population. The systematic examination of cognitive biases at different levels of information processing and for both types of content is important for making systematic statements not only regarding the presence but also the magnitude and specificity of cognitive biases in patients with AN.

An additional limitation of the previous literature is that most studies on cognitive biases in AN have been performed in adult populations. Regarding interpretation and memory biases, no studies to date have compared adolescents with AN to healthy adolescents.^1^ However, the transfer of results on cognitive biases obtained in studies on adults to adolescents is problematic as major cognitive and affective development is ongoing during adolescence [35–37]. Therefore, dysfunctional cognitions might either play a smaller role in adolescent than adult AN as cognitive patterns might not have evolved into stable, trait-like “cognitive styles” yet [as suggested for depression, e.g., 38]. Alternatively, young people might be particularly susceptible to negative cues in ambiguous emotional information due to brain maturation and hormonal changes associated with an enhanced emotional sensitivity [see e.g., 39], resulting in more pronounced cognitive biases. Considering also that the incidence of AN is highest during adolescence [e.g., 3], it is particularly relevant to investigate which dysfunctional cognitions are already present at this relatively early stage of the disorder. Cognitive biases are not only suggested to be involved in the development and maintenance of AN [e.g., 8, 9], but can also be experimentally manipulated in adults [e.g., 40–43] as well as adolescents [e.g., 34] with AN or other EDs. Therefore, knowledge about cognitive biases in adolescents with AN may help to develop interventions that step in early in the course of the illness, change underlying cognitive vulnerabilities before the illness takes a chronic course, and optimize existing therapies. Therefore, the aim of the present study is to systematically investigate whether adolescents with AN show cognitive biases in attention, interpretation, and memory and to examine to what extent these biases are specific for ED-related information and to what extent they are general negative biases (content-specificity).

When studying cognitive biases in adolescents with AN it should be taken into account that a high proportion [between 47 and 60%; 44, 45] of patients also suffer from comorbid mental disorders, especially depression and anxiety disorders [e.g., 44, 46]. Both depression [47, 48] and anxiety disorders [47] are associated with negative cognitive biases in adolescents, so the presence of negative biases for non-ED-related information in adolescents with AN might be explained by their comorbidities. On the other hand, negative biases for body-related stimuli may not be specific to patients with EDs, but may be an indicator of poor self-esteem which can be found transdiagnostically in various disorders [49]. In order to investigate to what extent biases are specific to individuals with AN (disorder-specificity), AN patients are not only compared with a healthy control group (HC group) but also with a clinical control group (CC group) consisting of patients with major depression or particular anxiety disorders.

The present study assesses attention, interpretation, and memory biases within a single experimental paradigm [see 50] based on the SST [51]. The SST was initially designed as a paper–pencil test to assess depression-specific interpretation biases [51] but was converted and extended by Everaert et al. [50] into a computerized paradigm including eye-tracking that allows additional assessment of attention and memory biases. Participants have to form sentences out of arrays of words. In each trial, there is a positive and negative target word that can be used to build a positive or a negative sentence. Attention biases are derived from dwell times on negative vs. positive target words assessed via eye-tracking while interpretation biases are derived from the behavioural outcome, i.e., the number of negatively versus positively resolved sentences. Memory biases are measured via free recall of the sentences at the end of the experimental session. Thus, within the same task, i.e., using the same stimulus material, biases on all three levels of information processing can be assessed. As both positive and negative information are included within each trial, the SST measures biases for negative versus positive information, i.e., relative biases. The SST has already been used to assess depression-related biases in adolescent samples [e.g., 52–55] and found to be a valid and reliable measure, particularly for the assessment of interpretation biases [split-half reliability acceptable to excellent; 54, 55]. It assesses automatic and implicit aspects of attention, interpretation, and memory biases [e.g., 54], thereby being less susceptible to problems typical for self-report measures that allow answers to be influenced by demand characteristics, social desirability, response biases, or deliberate response strategies [e.g. 56, 57]. Biases for ED-related information and biases for non-ED-related information are assessed with different types of stimuli within the SST. Stimuli related to one’s body, weight, and shape are used to assess ED-related cognitive biases. We focus on body-related instead of food- or eating- related stimuli as implicit measures using body stimuli were found to better discriminate between females with and without eating disorders than measures using food stimuli [58]. Stimuli related to social rejection or non-body- or -appearance-related self-devaluation are used to assess non-ED-related cognitive biases. These types of stimuli have frequently been used in studies assessing cognitive biases in depression and anxiety [see, e.g., 59–61] and also by some of the above mentioned studies investigating biases for non-ED-related information in AN [22, 29].^2^ Thus, within the same task biases on all three levels of information processing and across both types of content are systematically assessed, resulting in a 3 × 2 matrix of biases.

In line with findings in adult AN patients it is hypothesized that adolescents with AN will show more pronounced negative cognitive biases compared to healthy adolescents (HC group) for both ED-related information (hypothesis 1a) [e.g., 11, 12, 20] and non-ED-related information (hypothesis 2a) [e.g., 22, 29]. Furthermore, it is expected that adolescents with AN will show stronger biases for ED-related information than adolescents with depression or anxiety disorders (hypothesis 1b), whereas biases for non-ED-related stimuli will be similar or less pronounced in adolescents with AN compared to adolescents with depression or anxiety disorders (hypothesis 2b). Adolescents with depression or anxiety disorders (CC group) are not expected to differ from the HC group regarding ED-related biases (hypothesis 1c) but to show more pronounced negative biases for non-ED-related information than the HC group as well (hypothesis 2c) [in line with e.g., 47, 48]. In summary, for ED-related biases we expect the following pattern: AN > CC = HC (hypothesis 1) and for non-ED-related biases we expect: CC ≥ AN > HC (hypothesis 2). Thus, we expect specificity of biases to translate into differential results for the two types of content (i.e., Group × Content interactions in the analyses). In addition, we will explore to what extent cognitive biases are associated with participants’ psychopathology.

## Methods

This is an experimental cross-sectional study comparing adolescents with AN to a HC group as well as a CC group on measures of ED-specific and non-ED-specific cognitive biases. The study has received ethical approval by the ethics committee of the Medical Faculty of the LMU Munich (Project-No. 20-480). Written informed consent is obtained from all participants and—for participants younger than 18 years—also from their parents/legal guardians after a comprehensive explanation of the procedures.

### Participants

A total of 105 girls aged 12–18 years will be included in the study: 35 patients with AN, 35 clinical controls (patients suffering from a depressive episode or an anxiety disorder; CC group) and 35 healthy controls (HC group). Due to the gender imbalance with significantly higher prevalence of AN in women than in men [gender ratio female:male approximately 10:1; e.g., 3], only female adolescents are included. Participants are included in the AN group if they meet diagnostic criteria for AN according to DSM-5 [1]. Participants are included in the CC group if they meet criteria for major depression, social phobia, or generalized anxiety disorder according to DSM-5 [as these are the most frequent comorbidities of AN; e.g., 44, 46; and at the same time known to be associated with cognitive biases; e.g., 47] and do not meet criteria for an ED currently or in the past. Participants are included in the HC group if they do not meet criteria for any current or past mental disorder (axis 1 disorders as assessed with a standardized diagnostic interview, see below). Exclusion criteria for all groups are: below average intelligence [IQ < 85; assessed with the CFT 20-R; 62], insufficient German language skills, non-corrected visual impairment, pervasive developmental disorders, psychotic disorders, bipolar disorder, or substance abuse.

#### Sample size

As research on cognitive biases in adolescents with AN is very scarce we had to rely on results in adult AN samples for our sample size calculation [via G*Power; 63]. The aim of the present study is to investigate whether adolescents with AN show attention, interpretation, and memory biases. With regard to attention biases for ED-related information (i.e., body-related stimuli), a meta-analysis [15] reported a medium-sized effect (Glass' *g* = 0.5) for the group difference between patients with AN and HCs. Regarding attention biases for non-ED-related information, studies reported small [*d* ≤ 0.3; 26–28, 31, 32] to large [*d* ≥ 1.1; 23, 24] effects. With respect to interpretation biases, large effects (*d* ≥ 1.5) were reported for ED-specific [12, 18] as well as non-ED-specific biases [29]. A large effect was also reported for ED-specific memory biases [*d* = 0.8; 21]. In order to detect medium to large (*η*_*p*_^2^ = 0.08)^3^ main effects of the factor Group with an α of 0.05 and a power of 0.80 in a multi-factorial analysis of variance (ANOVA) with three groups, a sample size of *N* = 87 subjects is required. Furthermore, we want to examine to what extent these cognitive biases are specific for ED-related information and expect specificity of biases to translate as Group × Content interactions in the multi-factorial ANOVAs. None of the previous studies reported an effect size for such an interaction. If a small to medium-sized effect is assumed (*η*_*p*_^2^ = 0.03), a sample size of *N* = 81 subjects is necessary to detect the interaction. Since effects might be smaller in adolescent patients and experience shows that some subjects will have to be excluded from the analysis due to insufficient data quality in the experimental paradigms that we apply (see below for criteria), we aim for a total sample size of at least 105 subjects, *n* = 35 per group to ensure sufficient power.

#### Recruitment

Participants in the AN and CC groups are recruited through the Department of Child and Adolescent Psychiatry, Psychosomatics and Psychotherapy of the LMU University Hospital Munich. Potentially eligible in- and outpatients and their parents/legal custodians are approached by a member of the study team and provided a comprehensive explanation of the study procedures. Participants in the HC group are recruited via previous studies in which they had participated as healthy controls. Families receive a letter with information about the study and an invitation to participate. Participants receive a reimbursement of €30 as shopping vouchers (e.g., for Amazon).

#### Psychopathology assessment and self-report data

All participants undergo extensive diagnostic assessment before inclusion in the study. A standardized, semi-structured diagnostic interview [Kinder-DIPS; 65, 66] is conducted to assess psychiatric diagnoses. The Kinder-DIPS is a well-established German diagnostic interview that allows diagnosis of a wide range of psychiatric axis I disorders according to DSM-5 [1] with good interrater-reliability [66]. The interviews are conducted and evaluated by trained interviewers. Interrater-reliability will be determined for 25% of the participants by an independent researcher re-rating audio recordings of the diagnostic interviews (pre-defined criteria: lifetime-diagnoses of AN, major depression, social phobia, or generalized anxiety disorder). In the AN and CC groups the diagnostic interview serves to confirm diagnoses of AN, major depression, or anxiety disorders and to detect comorbidities. In the HC group the interview helps to rule out the presence of any current or past mental disorders. In addition, ED symptoms are assessed using the Eating Disorder Inventory [EDI-2; 67], depressive symptoms are assessed using the Beck Depression Inventory [BDI-II; 68], and the anxiety symptoms are measured using the State-Trait Anxiety Inventory [STAI; 69]. Height and weight (to calculate body mass index, BMI) of AN patients and CC inpatients are obtained from their physicians while HCs and CC outpatients are weighed and measured in our laboratory. Furthermore, sociodemographic data, self-esteem [Rosenberg Self-Esteem Scale, RSES; 70], body dissatisfaction [Body Shape Questionnaire, BSQ; 71], and social appearance anxiety [Social Appearance Anxiety Scale, SAAS; 72, 73] are assessed via questionnaires.

### Study procedure

For participating adolescents, the study consists of two sessions. In the first session, the diagnostic assessment takes place, i.e., IQ is assessed with the CFT 20-R [62] and the diagnostic interview [Kinder-DIPS; 65, 66] is conducted. If the adolescent is eligible for inclusion in the study, she is invited to the second session that takes place approximately one week later. In that session, cognitive biases are assessed using an experimental paradigm. Between the two sessions, participants are asked to fill out some of the questionnaires (sociodemographic questionnaire, EDI-2, Trait Version of the STAI, RSES, BSQ, SAAS). The remaining questionnaires (BDI-II, State Version of the STAI) are filled out at the beginning of the experimental session.

### Experimental paradigm

Cognitive biases are measured with a computerized version of the SST [adapted from 50, 54]. This task was originally designed to assess interpretation biases [51] but is administered during eye-tracking in the present study to enable simultaneous assessment of attention biases. It is followed by an incidental free recall test of the previously constructed interpretations that assesses memory biases [cf. 50].

The task consists of three types of trials: (1) ED-related (EDR) emotional trials that allow the assessment of cognitive biases for ED-related information, (2) non-ED-related (NED) emotional trials that allow the assessment of non-ED-specific cognitive biases, and (3) neutral trials that will not be analyzed.

#### Stimuli

Stimuli consist of 70 scrambled sentences: 28 EDR emotional sentences (e.g., “my fat bottom find attractive I”), 28 NED emotional sentences (e.g. “total I winner a loser am”), and 14 neutral sentences (e.g., “I exciting watching funny movies like”). All sentences contain six words and have two possible solutions. Emotional sentences include one positive target word (“attractive”, “winner”) which can be used to build a positive solution (“I find my bottom attractive”, “I am a total winner”) and one negative target word (“fat”, “loser”) which can be used to build a negative solution (”I find my bottom fat”, “I am a total loser”). The EDR emotional stimuli are based on the stimulus set developed by Brockmeyer et al. [12], which was adapted and extended. They consist of self-referent sentences related to the evaluation of one’s body and physical appearance. The NED emotional stimuli are based on the original stimulus set developed by Wenzlaff and Bates [51], which was translated into German [74], extended, and adapted. They consist of self-referent sentences involving self-evaluation that is not related to body, weight, or appearance. A similar stimulus set has already used in previous studies in youth [54, 55]. Neutral sentences are also self-referent but do not involve any positive or negative evaluation of oneself. As such, both solutions are emotionally neutral. The stimuli are constructed in such a way that they include no negations and the target words are emotional words corresponding in valence to the respective solution (i.e., positive words correspond to positive solutions and negative words correspond to negative solutions). Target words are matched for length and frequency in the German language across the stimulus set.^4^ In line with Everaert et al. [50], word position within each sentence is randomized with target words not allowed next to each other or in the first or last position and counterbalanced whether the positive or negative target word is presented first.

#### Procedure

**Fig. 1:**
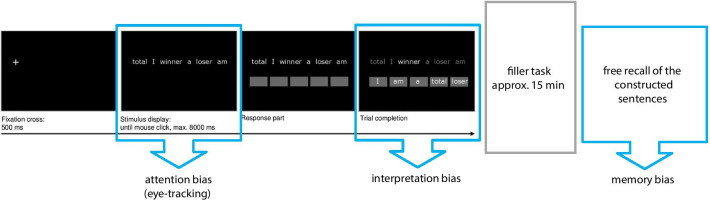
Example of a NED emotional SST trial and overview of the experimental procedure

The trial procedure is depicted in Fig. 1. The experiment is presented using Experiment Builder 1.10 [75]. Each trial starts with a fixation cross presented for 500 ms on the left side of the screen. After that, the stimulus display appears, consisting of six words in scrambled order presented at the center of the screen on a single line. Stimuli are presented in white Arial font size 20 on black background. Participants are instructed to read the words, mentally form a grammatically correct five-word sentence as quickly as possible, and click on the mouse button as soon as they did so to continue to the response part of the trial. The scrambled sentence is presented for a maximum of 8000 ms; if no mouse click occurs during that time the response part is omitted and the next trial begins. In the response part, five boxes appear below the scrambled sentence and participants are required to build the sentence they had mentally formed by ordering the words into the five boxes via mouse click.

Trials are randomly divided into seven blocks of ten, each containing four ED-related emotional trials, four non-ED-related emotional trials, and two neutral trials. Trials are presented in a random order within blocks and blocks are also presented in a random order. Before the first block, participants complete five neutral practice trials to familiarize themselves with the task. Similarly to earlier studies [e.g., 50, 52] a cognitive load procedure is included to prevent deliberate response strategies: Before each block, a 5-digit number is presented for 5000 ms which has to be memorized and recalled at the end of the block.

After the SST a filler task (an unrelated task consisting of watching pictures) lasting approximately 15 min is administered [e.g., 76]. Filler tasks serve to displace the content from the working memory so participants have to retrieve the content from episodic memory during a subsequent free recall [77]. Afterwards an incidental free recall test is administered. Participants are asked to recall as many of the sentences they had previously formed as possible and write them on a piece of paper. Up to ten minutes are allowed for this task but the participants are not informed about the time limit.

Attention biases are assessed via eye movement registration during the stimulus display parts of the trials while interpretation biases are derived from the behavioural results, i.e., the sentences the participants construct in the task. Memory biases are measured with the free recall test at the end of the experimental paradigm (see Fig. 1).

#### Eye-tracker

Eye movements are registered with an EyeLink 1000 Plus Desktop mounted eye-tracker which uses infrared video-based tracking technology [78]. Participants are seated in front of a 15-inch monitor (1024 × 768 pixel resolution) on which the experiment is presented. Viewing is binocular while eye movements are registered from the dominant eye with a sampling rate of 1000 Hz. A forehead and chin rest are used to minimize head movements and keep the viewing distance constant at 65 cm. Lighting of the room is kept constant for all participants. Before the task starts, a 9-point calibration and validation procedure is conducted and calibration is accepted if the average error is less than 0.5° of visual angle and the maximum error is less than 1° of visual angle.

Eye movement events are detected using a velocity- and acceleration-based saccade detection method with saccades defined, in line with previous studies [e.g., 79–81], as events with a velocity above the threshold of 30°/s or an acceleration above the threshold of 8000°/s^2^. Gaze positions that are stable within 1° of visual angle for at least 60 ms are defined as fixations [in line with e.g., 82, 83].

### Analyses

#### Data processing and outcome variables

Participants’ responses (i.e., the sentences they built in the SST and the sentences they remembered in the free recall test) will be rated as correct or incorrect according to predefined criteria by two independent raters. Trials in which no grammatically correct sentence is built (time-out or incorrect sentence) will be excluded from the analyses. Participants with a correct sentence rate of three standard-deviations (*SD*) below the mean (in the SST) will be identified as outliers in terms of accuracy [e.g., 55] and excluded from all analyses.

For analysis of attention biases, we will additionally exclude trials with poor eye-tracking data quality [i.e., trials in which the total dwell time is less than 75% of the presentation time due to excessive blinks, missing data, or participants not looking at the screen; 79, 80]. Subsequently, participants with insufficient remaining trials [less than 75%; e.g., 80] and participants with systematic calibration errors (identified by visual inspection) will be excluded from the analysis of attention biases. Attention bias scores will be calculated by dividing the percentage of dwell time on negative target words (i.e., on the areas of interest around those words) by the sum of percentage of dwell times on negative and positive target words, so that a higher value indicates a more negative attention bias [similar to 50]. Separate scores for EDR and NED sentences will be calculated, resulting in an AB_EDR_ and an AB_NED_ score.

For analysis of the interpretation bias, the correctly unscrambled emotional sentences will be categorized as either positive or negative. Two interpretation bias scores will be calculated: IB_EDR_ reflects the proportion of negatively resolved sentences from the total number of correctly resolved EDR sentences while IB_NED_ reflects the proportion of negatively resolved sentences from the total number of correctly resolved NED sentences [50].

For analysis of memory biases, memory bias scores (MB_EDR_ and MB_NED_) will be calculated similarly: as the proportion of correctly remembered negative sentences from the total number of correctly remembered EDR or NED sentences [50], respectively.

Thus, two relative bias scores emerge on each level of information processing: one reflecting bias for *negative versus positive ED-related information* (AB_EDR_, IB_EDR_, and MB_EDR_) and one reflecting bias for *negative versus positive non-ED-related emotional information* (AB_NED_, IB_NED_, and MB_NED_).

#### Reliability

Split-half reliability of the bias scores will be assessed by correlating bias scores based on odd versus even trials [see e.g., 54, 55, 84]. Importantly, only bias scores with at least acceptable reliability (Spearman-Brown corrected reliability > 0.7) will be analyzed and reported. In addition, to rule out that the results are influenced by the subjective rating of the participants’ responses, interrater-reliability will be determined for IB and MB bias scores.

#### Analyses of hypotheses

Statistical analysis of the data will be performed using IBM SPSS Statistics. Group differences in demographic and clinical characteristics (age, IQ, body-mass-index as well as ED, depression, and anxiety symptoms) and other questionnaire scores (self-esteem, body dissatisfaction, and social appearance anxiety) will be examined using univariate ANOVAs with the between-subjects factor Group (3: AN, CC, HC) and follow-up *t*-tests. The hypotheses will be addressed with separate analyses for attention, interpretation, and memory biases. Repeated-measures ANOVAs on bias scores will be performed with within-subject factor Content (2: EDR, NED) and between-subjects factor Group (3: AN, CC, HC). Significant main effects and interactions will be followed up by post-hoc ANOVAs and *t*-tests. We expect our hypotheses (hypothesis 1 regarding ED-related biases: AN > CC = HC; hypothesis 2 regarding non-ED-related biases: CC ≥ AN > HC) to translate as Group × Content interactions in the ANOVAs and corresponding results in the post-hoc tests. Associations between bias scores and psychopathology (questionnaire measures) will be assessed via Pearson’s correlations. For all analyses, the significance level will be set to *p* = 0.05 (two-tailed) and adjusted according to the Bonferroni–Holm procedure [85] when multiple post-hoc comparisons are performed. Effect sizes will be reported for all significant effects: *η*_*p*_^2^ and *η*_*G*_^2^ for ANOVA effects and Cohen’s *d*_*s*_ for between-group *t*-tests, as suggested by Lakens [86]. In addition to testing our hypotheses, we will explore to what extent cognitive biases are related to participants’ psychopathology by calculating correlations between bias scores and ED, depression, and anxiety symptoms as well self-esteem, body dissatisfaction, and social appearance anxiety.

## Discussion

Dysfunctional cognitions including cognitive biases are known to characterize individuals with AN. However, less is known about the specificity of biases, i.e., if biases in AN patients occur only for ED-related information or also for non-ED-related emotional information (content-specificity) and if biases are unique to individuals with AN or are also shown by individuals with other mental disorders (disorder-specificity). The present study is designed to systematically assess cognitive biases in adolescents with AN on three levels of information processing (attention, interpretation, and memory) and with regard to two types of information (ED-related and non-ED-related information). To address not only content- but also disorder-specificity, adolescents with AN are compared not only to a healthy but also to a clinical control group [comprising adolescents with major depression, social anxiety, or generalized anxiety disorder, which are the most frequent comorbidities of AN; e.g., 44, 46].

We expect to find more pronounced negative cognitive biases on all three levels of information processing and for both types of content in adolescents with AN compared to healthy adolescents [in line with studies in adults; e.g., 11, 12, 20, 22, 29]. We expect the specificity of biases to translate as differential results for the two types of content with the AN group showing stronger biases for ED-related information but similar or less pronounced biases for non-ED-related information than the group of CCs [who are expected to show more pronounced biases for non-ED-related information; see 47, 48; but similar biases for ED-related information in comparison to the HC group].

The study focuses on 12–18-year-old adolescent girls as adolescence is the most common time for the onset of AN [e.g., 3] while also being a time in which major cognitive and affective development is ongoing [e.g., 37], making it difficult to transfer results obtained in adult populations on this age group. Insights about factors contributing to the maintenance of AN already at a relatively early stage may be particularly informative for interventions that step in early in the course of the disorder.

Major strengths of the study include a thorough diagnostic assessment of all participants, the systematic and comprehensive assessment of cognitive biases addressing multiple levels of information processing as well as the content-specificity hypothesis, and the inclusion of a clinical control group to also address the question of disorder-specificity. Furthermore, an elaborate data processing and analysis strategy has been decided upon beforehand.

Influential publications have underlined the importance of experimentally investigating key processes of psychopathology in EDs [87] and especially AN [88] as this may shed light on the mechanisms that cause and maintain the disorder as well as the mechanisms that need to be targeted in order to change and reduce ED psychopathology. Cognitive biases may be a promising starting point since they are both, presumably involved in the etiology and maintenance of AN [e.g., 8–10], as well as modifiable in experimental settings [e.g., 34, 40, 41, 89–91]. However, as also suggested by Paslakis and colleagues [57], precisely specifying what kinds of cognitive biases characterize patients with AN and investigating the extent to which these biases are related to their psychopathology is an essential precursor for efficiently experimentally modifying biases in this particular population. As attempting to manipulate cognitive biases only makes sense for biases that (1) characterize the target population (in comparison to control groups), (2) are related to their psychopathology, and (3) can be reliably measured, systematic knowledge on biases can help subsequent studies to choose the most promising ones as their interventional targets.

In summary, the present study, which is the first to assess cognitive biases in AN in such a comprehensive way, particularly in adolescents, is likely to advance our knowledge about an important maintaining mechanisms of AN and make a valuable contribution to the field. It will have important implications for cognitive behavioural theories of AN as well as subsequent studies aiming to modify cognitive biases in adolescent AN patients.^5^

## Study status

The KOALA-study is ongoing. Data collection started in September 2020 and will continue approximately until December 2021.

## Data Availability

Data collection for the study is still on-going. In order to support open science, data will be made publicly available via the Open Science Framework. Data that is collected will also be available to share with other researchers upon reasonable request.

## Abbreviations

AB_EDR_: Attention bias for eating disorder-related information
AB_NED_: Attention bias for non-eating disorder-related information
AN: Anorexia nervosa
ANOVA: Analysis of variance
BDI-II: Beck depression inventory-II
BMI: Body mass index
BSQ: Body shape questionnaire
CC: Clinical control
CFT 20-R: Culture fair test 20 revision
Kinder-DIPS: Diagnostisches Interview bei psychischen Störungen im Kindes-und Jugendalter
DSM-V: Diagnostic and statistical manual of mental disorders, fifth edition
ED: Eating disorder
EDI-2: Eating disorder inventory 2
EDR: Eating disorder-related
HC: Healthy control
IB_EDR_: Interpretation bias for eating disorder-related information
IB_NED_: Interpretation bias for non-eating disorder-related information
IQ: Intelligence quotient
MB_EDR_: Memory bias for eating disorder-related information
MB_NED_: Memory bias for non-eating disorder-related information
NED: Non-eating disorder related
RSES: Rosenberg self esteem scale
SAAS: Social appearance anxiety scale
SST: Scrambles sentences task
STAI: State-trait anxiety inventory

## Footnotes

1 The only study that assessed interpretation biases in adolescents with AN reported a negative non-ED-specific bias in the adolescent sample, but did not include a control group [34].

2 An additional benefit of using body-related (instead of food- or eating-related) stimuli for assessing ED-related cognitive biases is that we were able to generate similar stimuli to assess ED-related and non-ED-related biases: both types of stimuli are self-referent and involve a positive and a negative evaluation of oneself but differ in the involvement of body/physical appearance evaluation. Additional details are provided in the methods section.

3 According to Cohen [64], *η*_*p*_^*2*^ = .01 corresponds to a small effect, *η*_*p*_^*2*^ = .06 corresponds to a medium effect, and *η*_*p*_^*2*^ = .14 corresponds to a large effect.

4 EDR trials: Positive target words: Word length *M* = 8.3 (*SD* = 2.2) characters, word frequency (category according to http://wortschatz.uni-leipzig.de/de) *M* = 13.8 (*SD* = 2.7); Negative target words: word length *M* = 7.8 (SD = 2.8), word frequency *M* = 15.1 (*SD* = 3.0). NED trials: Positive target words: Word length *M* = 8.8 (*SD* = 2.1) characters, word frequency *M* = 11.8 (*SD* = 2.9); Negative target words: word length *M* = 8.7 (*SD* = 2.3) characters, word frequency *M* = 12.8 (*SD* = 2.4); *t*s ≤ 1.7, *p*s > .05.

5 It has to be noted, however, that finding particular cognitive biases to characterize AN patients and to be associated to their psychopathology is necessary but not sufficient for establishing biases as etiological or maintaining factors. Studies experimentally manipulating biases and studies investigating biases longitudinally will be a necessary next step to achieve this goal.
